# Relationship among physical activity, social anxiety, and autistic traits in female college students: a variable-and person-centered analysis

**DOI:** 10.3389/fpsyt.2026.1786160

**Published:** 2026-05-28

**Authors:** Biao Huang, Xinyi Chen, Chang Hu, Wenying Huang, Dong Zhu, Yan Liu, Huiran Feng, Bo Xu

**Affiliations:** 1 School of Physical Education, Jiangxi Normal University, Nanchang, China; 2 Hunan University of Medicine, Huaihua, China

**Keywords:** autistic traits, female college students, latent profile analysis, physical activity, social anxiety

## Abstract

**Background:**

Autistic traits are prevalent among college students and are associated with impaired social functioning and mental health. Although physical activity is considered a protective factor, the underlying mechanisms, particularly the mediating role of social anxiety and the heterogeneity of autistic traits, remain unclear. This study aimed to examine the association between physical activity and autistic traits, test the mediating role of social anxiety, and identify latent subgroups among female college students.

**Methods:**

A cross-sectional survey was conducted among 2,137 female college students (M_age = 19.31, SD = 1.17). Autistic traits, social anxiety, and physical activity were assessed using the Autism-Spectrum Quotient-10 (AQ-10), the Social Interaction Anxiety Scale-6 (SIAS-6) and the Social Phobia Scale-6 (SPS-6), and the Physical Activity Rating Scale-3 (PARS-3), respectively. Mediation analysis was performed using PROCESS (Model 4), and latent profile analysis (LPA) was conducted to identify subgroups.

**Results:**

Physical activity was significantly negatively associated with social anxiety (*β* = -0.205, *p* < 0.001) and autistic traits (*β* = -0.197, *p* < 0.001), while social anxiety was positively associated with autistic traits (*β* = 0.425, *p* < 0.001). Social anxiety partially mediated this relationship (indirect effect = -0.087, 95% CI [-0.106, -0.067]), accounting for 31% of the total effect. Latent profile analysis identified two profiles: low autistic traits (74.54%) and high autistic traits (25.46%). The high trait group showed lower physical activity (*t* = 7.91, *p* < 0.001, *d* = 0.39) and higher social anxiety (*t* = -23.25, *p* < 0.001, *d* = -1.16).

**Conclusions:**

Physical activity and social anxiety are key modifiable factors associated with autistic traits. Interventions should promote physical activity while reducing social anxiety, particularly for students with elevated autistic traits.

## 1 Introduction

Autistic traits are defined as autism-spectrum-like characteristics manifested in social interaction, communication styles, and behavioral interests, originating primarily from heightened sensitivity to uncertainty and evaluative cues in social contexts (1, 2). These traits are dimensional and widely distributed in the general population, including difficulties in social communication (e.g., reduced eye contact), atypical language use, cognitive inflexibility, and restricted interests (3, 4). It is important to distinguish autistic traits from autism spectrum disorder (ASD), a clinically diagnosed neurodevelopmental condition defined by the DSM-5 and characterized by significant functional impairment (5). Individuals with elevated autistic traits may not meet ASD diagnostic criteria but can still experience notable psychosocial challenges (6, 7).

Autistic traits are prevalent among youth and are particularly common in college students, with approximately 15%–30% exhibiting moderate-to-high levels and about 1%–2% reaching clinically relevant thresholds (8–11). These traits have been linked to increased academic pressure and developmental challenges during the college years (12, 13) and are associated with social anxiety, loneliness, and maladaptive behaviors (14, 15). Female college students warrant particular attention in this field. Compared with male college students, females with elevated autistic traits are more likely to exhibit subtler or camouflaged presentations, which may obscure identification while leaving associated internalizing problems, particularly social anxiety, insufficiently recognized (16). At the same time, the college period involves heightened interpersonal and identity-related demands, which may render female students with elevated autistic traits especially vulnerable to psychosocial difficulties (17).

Accordingly, it is important to examine modifiable factors associated with autistic traits in this population. Physical activity has been widely linked to improved psychological well-being and social functioning (18, 19). In contrast, social anxiety, defined as the fear of negative evaluation in social situations (20), may serve as a key explanatory mechanism. Therefore, the present study aimed to examine the associations among physical activity, social anxiety, and autistic traits in female college students, and to test whether social anxiety mediates the relationship between physical activity and autistic traits.

### 1.1 Physical activity and autistic traits

In recent years, physical activity has been widely recognized as a cost-effective and accessible approach for promoting psychological and behavioral health (21, 22). According to the World Health Organization, physical activity refers to any bodily movement produced by skeletal muscles that results in energy expenditure. In contrast, physical exercise is a structured and planned subset of physical activity (23, 24). The present study focuses on broader engagement in physical activity rather than solely structured exercise.

Existing research indicates that physical activity contributes to enhanced social functioning and psychological adaptation (25–28). Drawing on the Broaden-and-Build Theory, physical activity can elicit positive emotional experiences such as enjoyment and relaxation, thereby expanding attentional scope and enhancing cognitive flexibility, which may help alleviate cognitive rigidity and stereotyped patterns associated with autistic traits (29, 30). Meanwhile, the Distraction Hypothesis suggests that physical activity facilitates a shift of attention from internal rumination to external movement processes, thereby interrupting repetitive thought patterns (31, 32). In addition, Social Cognitive Theory (SCT) posits that emotional states play a central role in behavioral regulation (33). When individuals perceive social threat or anxiety, they are more likely to adopt avoidance behaviors. Physical activity may reduce such avoidance tendencies by improving emotional states and enhancing self-regulation capacity, thereby promoting more adaptive social engagement (34, 35).

At the contextual level, physical activity also provides a natural platform for social interaction, which may contribute to improved social adaptation (36). Empirical evidence suggests that participation in group-based physical activities can enhance social communication and adaptability among individuals with autistic traits, and regular physical activity is associated with better mental health and lower levels of internalizing problems (37). Longitudinal studies further indicate that physical activity predicts better psychological functioning and reduces behaviors such as social withdrawal, which substantially overlap with autistic traits (38–40).

### 1.2 The mediating role of social anxiety

Social anxiety may play a pivotal mediating role between physical activity and autistic traits. Social anxiety is typically defined as a persistent fear of negative evaluation in social or performance situations, accompanied by heightened distress and avoidance tendencies (16). Within the framework of Social Cognitive Theory (SCT), emotional states play a central role in behavioral regulation. When individuals experience elevated levels of social anxiety, they are more likely to adopt avoidance strategies, such as social withdrawal or reduced interaction, which are also characteristic features of autistic traits (41).

Physical activity may alleviate social anxiety through multiple pathways. Specifically, engaging in physical activity can enhance individuals’ sense of competence and provide positive social feedback, thereby reducing perceived social threat and promoting more adaptive emotional regulation (42–44). As social anxiety decreases, individuals experience less fear in social situations and improved self-regulation capacity, which manifests behaviorally as reduced social withdrawal and fewer stereotyped or avoidant responses, ultimately reflecting lower levels of autistic traits (45, 46).

A substantial body of empirical evidence supports these theoretical assumptions. Studies focusing on college student populations have shown that regular physical activity is associated with lower levels of social anxiety and better emotion regulation (47, 48). As a key psychological construct, social anxiety reflects individuals’ tendency toward tension and avoidance in socially evaluative contexts. It has been identified as a significant predictor of social withdrawal and maladaptive behaviors (49). Higher levels of social anxiety are associated with increased avoidance behaviors and interpersonal difficulties, which may exacerbate behavioral manifestations related to autistic traits (50–52). In contrast, lower levels of social anxiety are typically linked to better social adaptability and fewer autism-like behaviors (53). Although existing literature has addressed these variables individually, the specific intrinsic integrative mechanisms linking physical activity, social anxiety, and autistic traits warrant further systematic exploration.

### 1.3 Latent profile analysis

Existing scholarship has predominantly adopted a variable-centered perspective. However, such methodologies, which rely on sample averages, often fail to adequately capture inter-individual heterogeneity (54). By analyzing distinct subgroups of individuals, researchers can gain a deeper understanding of variations in physical activity and social anxiety, as well as how these differences influence autistic traits (55). Consequently, analyzing the latent categories of autistic traits from a person-centered perspective is of significant importance. Latent Profile Analysis (LPA) constitutes a person-centered statistical approach that clarifies the links among observed variables through latent continuous variables and further elucidates relationships among observed variables using latent class variables (56, 57).

In recent years, a limited number of studies have employed this methodology to explore latent categories of autistic traits. For instance, certain studies have identified two distinct types of autistic traits (58), while others suggest that a three-class model captures the nuances more effectively (59). Nevertheless, research on the latent categories of autistic traits remains relatively scarce, and classification results across studies have yet to reach consensus. This implies that a definitive consensus regarding the underlying structure and characteristics of autistic traits remains elusive. Based on current research, this study aims to further refine the latent patterns of autistic traits among female college students. Identifying distinct types of autistic traits facilitates a more profound grasp of the relationships between physical activity, social anxiety, and autistic traits of varying levels and characteristics. This approach allows for transcending the limitations of average effects to reveal internal sample heterogeneity, thereby providing a basis for formulating more targeted intervention strategies.

### 1.4 Current research

Synthesizing the aforementioned literature, the present research aims to investigate the potential inverse relationship between physical activity and autistic traits, while also exploring the mediating role of social anxiety in this association. Moreover, this research employs LPA to identify distinct latent profiles of autistic traits among female college students and to explore variations in physical activity and social anxiety among the profiles. This comprehensive analytical approach, combining both variable-centered and person-centered perspectives, not only enriches existing literature by elucidating the complex mechanisms underlying autistic traits but also offers novel translational perspectives for tailoring mental health interventions for female college students.

Building on relevant theoretical models and prior research, we formulate three key hypotheses: (H1) Physical activity is inversely and markedly associated with autistic traits. (H2) Social anxiety plays a significant mediating role in the relationship between physical activity and autistic traits. (H3) Autistic traits among female college students exhibit distinct latent profiles, and these profiles exhibit significant disparities in physical activity and social anxiety levels.

## 2 Materials and methods

### 2.1 Participants

The present study employed a cross-sectional survey design. Data were collected between September 10 and December 22, 2025, using offline paper-and-pencil questionnaires administered in classrooms and libraries across universities in China. To ensure adequate statistical power for the planned analyses, an *a priori* power analysis was conducted using G*Power 3.1 for the intended regression-based mediation model (60). The results indicated that, given a medium effect size (*f²* = 0.15), a significance level of α = 0.05, and a desired power of (1−*β*) = 0.95, a sample size of at least 124 participants is necessary to detect the target effect. The actual effective sample size obtained in this study (*N* = 2,137) substantially exceeded this threshold, thereby providing a robust statistical basis for subsequent model testing and interpretation.

A convenience sampling strategy was adopted to recruit participants from universities in China. Eligibility criteria were as follows: (a) biologically female; (b) currently enrolled as full-time undergraduate students; (c) no physical disabilities that prevent participation in physical activity; and (d) voluntary participation with informed consent. To ensure data quality, all returned questionnaires underwent manual verification, and the following exclusion criteria were applied: (a) self-reported history of clinically diagnosed psychiatric or neurological disorders (e.g., depression, schizophrenia, or clinically diagnosed autism spectrum disorder); (b) questionnaires with significant missing data; and (c) invalid questionnaires characterized by regular response patterns (e.g., straight-lining) or obvious inattentive responding.

All participants provided written informed consent before participation. They were fully informed of the study’s purpose and procedures, the voluntary nature of participation, and their right to withdraw at any time without penalty. The anonymity and confidentiality of all responses were strictly ensured, and no personally identifiable information was collected.

The study protocol was reviewed and approved by the Institutional Review Board of Jiangxi Normal University (IRB-JXNU-PEC-20240106). All procedures were conducted in accordance with the ethical standards of the Declaration of Helsinki.

### 2.2 Measurements

#### 2.2.1 Autistic traits scale

Autistic traits were measured using the 10-item Autism Spectrum Quotient (AQ-10) developed by Baron-Cohen (61). Comprising 10 items, this scale is widely employed for the rapid screening and measurement of adult autistic traits due to its brevity and ease of administration. Items are rated on a 4-point Likert scale (0 = definitely disagree, 3 = definitely agree), and reverse-scored items were recoded before statistical analysis. Total scores range from 0 to 30, with higher scores indicating more pronounced autistic traits. The widely used Chinese version of the AQ-10 was adopted in this study, which has demonstrated good applicability in previous studies involving Chinese university students (62). In the current study, the Cronbach’s α coefficient for this scale was 0.950.

#### 2.2.2 Social anxiety anxiety scale

Social anxiety levels were assessed using the short forms of the Social Interaction Anxiety Scale (SIAS-6) and the Social Phobia Scale (SPS-6), as revised by Peters (63). The instrument consists of 12 items across two subscales: social interaction anxiety (SIAS-6) and social phobia (SPS-6), each with 6 items. All items were rated on a 5-point Likert scale ranging from 0 (“not at all characteristic of me”) to 4 (“extremely characteristic of me”). Scores for each subscale range from 0 to 24, with higher scores indicating greater levels of social interaction anxiety or social phobia, respectively. The total social anxiety score is obtained by summing all items, with a possible range from 0 to 48, where higher scores reflect higher overall levels of social anxiety. The Chinese version of the SIAS-6/SPS-6 has been validated in college student populations and demonstrates good reliability and validity (64). In the current study, the Cronbach’s α coefficient for the scale was 0.926.

#### 2.2.3 Physical activity

Participants’ physical activity levels were evaluated using the Physical Activity Rating Scale-3 (PARS-3) developed by Liang (65). This scale consists of three distinct items designed to measure the Intensity, duration, and Frequency of exercise. The total score for physical activity was calculated using the formula: Physical Activity Score = Intensity × (Duration - 1) × Frequency. Scores range from 0 to 100, with higher scores indicating higher levels of physical activity. Tailored specifically to Chinese university students, this instrument has demonstrated robust reliability and validity across extensive empirical research (66). In the current study, the Cronbach’s α coefficient for this scale was 0.748.

#### 2.2.4 Control variables

Given that autistic traits and social anxiety in female university students may be contingent upon specific background factors, particularly those related to demographic characteristics (67), variables such as grade, place of residence, and family structure were identified. Consequently, grade, place of residence, and only-child status were included as control variables in the analysis. These were dummy coded as follows: place of residence (1 = urban, 2 = rural), only-child status (1 = non-only child, 2 = only child), and grade (1 = freshman, 2 = sophomore, 3 = junior, 4 = senior).

### 2.3 Data analysis

Data analyses were conducted using SPSS 26.0 and Mplus 8.3 statistical software (68, 69). Preliminary analyses included descriptive statistics and Pearson correlation coefficients for all variables. The normality of the data was assessed using skewness and kurtosis indices. When the absolute values of skewness were less than 2, and those of kurtosis were less than 7, the data were considered not to deviate substantially from normality (70). Given that the skewness of social anxiety slightly exceeded the recommended threshold, both Pearson correlation and Spearman rank-order correlation analyses were performed to ensure the robustness of the findings. To assess potential common-method bias in self-reported data, Harman’s single-factor test was conducted.

For the variable-centered analysis, the PROCESS macro (Model 4) in SPSS was utilized to examine the mediating mechanism of social anxiety in the relationship between physical activity and autistic traits. Grade, place of residence, and only-child status were included as covariates in the analysis. The bias-corrected nonparametric percentile Bootstrap method (with 5,000 resamples) was used to estimate the 95% confidence interval (CI) for the mediation effect; a CI that does not contain zero indicates statistical significance.

For the person-centered analysis, latent profile analysis (LPA) was conducted using Mplus 8.3. Model fit was evaluated using the Akaike Information Criterion (AIC), the Bayesian Information Criterion (BIC), and the adjusted BIC (aBIC), with lower values indicating better fit. Entropy values were used to assess classification accuracy, with higher values indicating better classification. The optimal number of profiles was determined based on the Lo–Mendell–Rubin likelihood ratio test (LMR-LRT) and the bootstrap likelihood ratio test (BLRT), with statistical significance set at *p* < 0.05. A minimum class size of 5% was required to ensure interpretability (71). For group comparisons between latent profiles, independent-samples t-tests were conducted. To account for potential deviations from normality, Mann–Whitney U tests were also performed as a robustness check. Effect sizes were reported using Cohen’s d for t-tests and rank-biserial correlation for Mann–Whitney U tests where appropriate. Across all statistical analyses, a two-tailed p-value of < 0.05 was adopted as the criterion for statistical significance, unless otherwise specified.

## 3 Results

### 3.1 General information about respondents

As shown in **Table 1**, the mean age of the participants was 19.31 years (SD = 1.17). In terms of academic year, the sample was predominantly composed of sophomores (38.20%) and freshmen (34.10%), followed by juniors (22.20%) and seniors (5.50%). Most participants were only children (72.60%), and a greater proportion of the sample came from rural areas (59.00%) compared to urban areas (41.00%).

**Table 1:** Demographic characteristics of the participants (N = 2,137).

Variable	Category	*n (%)*	*M ± SD*
Age (years)	—	—	19.31 ± 1.17
Academic year	Freshman	728 (34.10)	—
	Sophomore	816 (38.20)	—
	Junior	475 (22.20)	—
	Senior	118 (5.50)	—
Only-child status	Only child	1,551 (72.60)	—
	Non-only child	586 (27.40)	—
Place of origin	Urban	876 (41.00)	—
	Rural	1,261 (59.00)	—

### 3.2 Common method bias test

To assess common method bias, Harman’s single-factor test was conducted. As shown in **Table 2**, the results indicated that four factors with eigenvalues greater than 1 were extracted. The first unrotated factor accounted for 41.23% of the total variance, which is below the commonly accepted threshold of 50% and close to the more conservative 40% criterion (72). These findings suggest that common method bias is unlikely to pose a serious threat to the validity of the results. In addition, procedural remedies, such as ensuring anonymity and voluntary participation, were implemented to further mitigate potential common method bias.

**Table 2:** Total variance explained from Harman’s single-factor test.

Component	Eigenvalue	% of Variance	Cumulative %
1	10.307	41.227	41.227
2	3.481	13.923	55.150
3	1.744	6.978	62.127
4	1.071	4.285	66.413

Only factors with eigenvalues greater than 1 are presented. Extraction method: Principal Component Analysis.

### 3.3 Correlational analysis

**Table 3** summarizes the descriptive statistics and correlation coefficients for physical activity, social anxiety, and autistic traits. Most variables exhibited acceptable levels of skewness (|skewness| < 2) and kurtosis (|kurtosis| < 7) (70). However, the skewness of social anxiety slightly exceeded the recommended threshold. Therefore, Spearman’s rank-order correlation was conducted as the primary analysis, while Pearson correlation analysis was additionally performed as a robustness check. The results indicated that physical activity (*M* = 42.30, *SD* = 35.93) was significantly negatively correlated with social anxiety (*r* = -0.378, *p* < 0.01) and autistic traits (*r* = -0.401, *p* < 0.01). Furthermore, social anxiety (M = 11.44, SD = 8.57) was significantly positively correlated with autistic traits (*r* = 0.566, *p* < 0.01). In addition, Pearson correlation analysis was conducted as a robustness check, and the results are presented in **Table 4**. The Pearson correlation results showed the same directional patterns and statistical significance as the Spearman correlations, further supporting the stability and reliability of the findings.

**Table 3:** Descriptive statistics and Spearman rank-order correlations among variables (*N* = 2137).

Variable	*M*	*SD*	Skewness	Kurtosis	1	2	3
1. Physical activity	42.30	35.93	0.641	-0.755	1		
2. Social anxiety	11.44	8.57	2.052	5.179	-0.378**	1	
3. Autistic traits	7.96	7.710	1.072	0.479	-0.401**	0.566**	1

**p < 0.01.

**Table 4:** Pearson correlations among variables (*N* = 2,137).

Variable	1	2	3
1. Physical activity	1		
2. Social anxiety	-0.208**	1	
3. Autistic traits	-0.293**	0.489**	1

**p < 0.01.

### 3.4 Variable-Centered centered analysis

**Figure 1:**
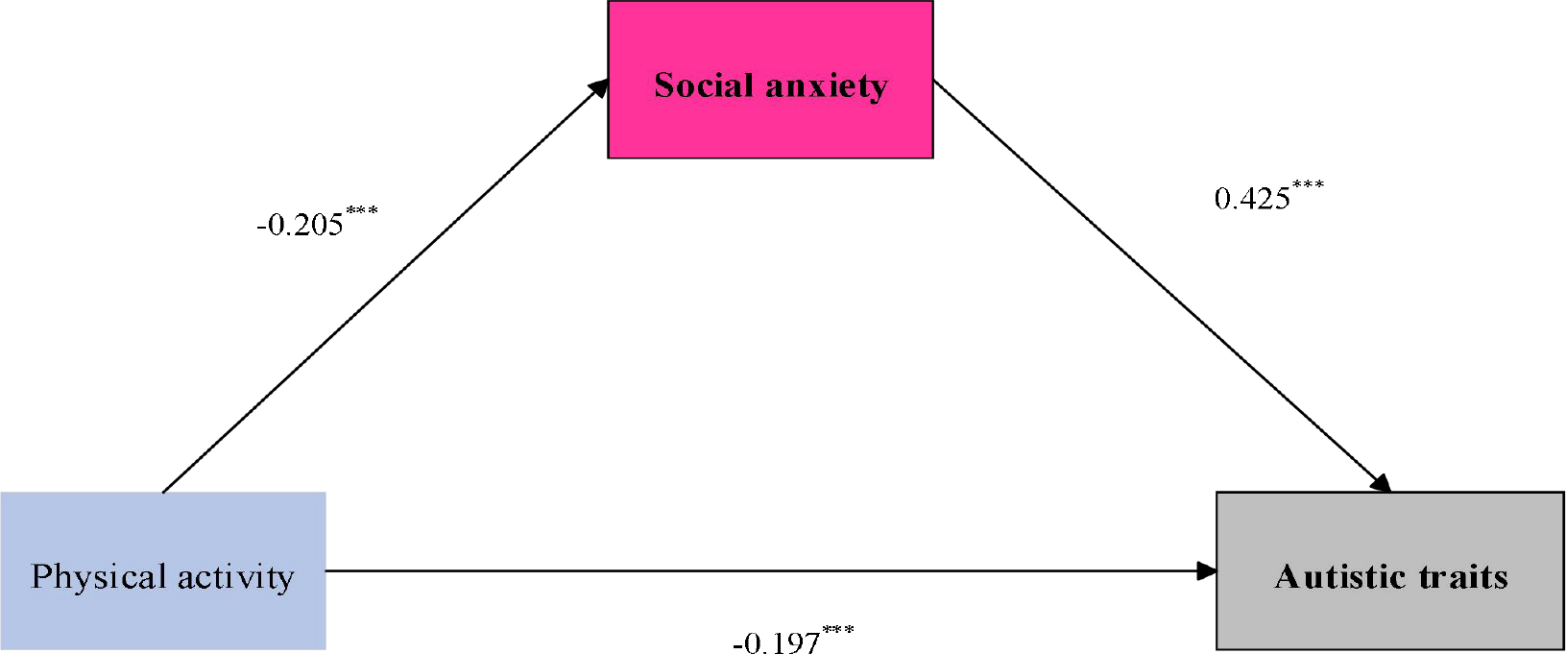
Path diagram of the mediation model. ***p < 0.001.

Hayes’ PROCESS macro (Model 4) was employed to test whether social anxiety mediated the association between physical activity and autistic traits. Origin, only-child status, and grade were included as control variables. The findings indicated a significant negative association between physical activity and social anxiety (*β* = -0.205, *p* < 0.001). When both physical activity and social anxiety were entered into the regression model predicting autistic traits, social anxiety demonstrated a significant positive association with autistic traits (*β* = 0.425, *p* < 0.001). Importantly, the direct effect of physical activity on autistic traits remained statistically significant and negative (*β* = -0.197, *p* < 0.001). This pattern suggests that social anxiety serves as a partial mediator of the association between physical activity and autistic traits (see **Table 5**, **Figure 1**).

**Table 5:** Testing the mediation model.

Variable	Social anxiety		Autistic traits	
	*β*	*t*	*β*	*t*
Place of Residence	0.148	3.498***	0.258	6.952***
Only Child Status	-0.417	-8.972***	-0.134	-3.229**
Grade	0.004	0.173	-0.004	-0.174
Physical activity	-0.205	-9.813***	-0.197	-10.553***
Social anxiety			0.425	22.395***
*R^2^*	0.084		0.296	
*F*	48.715***		179.516***	

The dependent variable is autistic traits; the independent variable is physical activity; the mediating variable is social anxiety; and the control variables are place of residence, only child status, and grade. **p < 0.01, ***p < 0.001.

The mediation effect was further evaluated using a Bootstrap procedure with 5,000 resamples. As reported in **Table 6**, the analysis revealed a significant indirect effect of social anxiety on the relationship between physical activity and autistic traits, with an effect estimate of -0.087 (95% CI: -0.106 to -0.067). Since the 95% confidence interval did not include “0,” the mediating effect is statistically significant. The mediated (indirect) pathway explained 31% of the overall effect. These results offer empirical evidence that social anxiety constitutes a key mechanism linking physical activity to autistic traits.

**Table 6:** Results of mediation effect analysis.

Path	Effect	BootSE	BootLLCI	BootULCI	Relative proportion
Total effect	-0.284	0.020	-0.324	-0.244	100%
Direct effect	-0.197	0.019	-0.234	-0.160	69%
Indirect effect	-0.087	0.010	-0.106	-0.067	31%

### 3.5 Latent profile analysis

Employing a person-centered perspective, the study further explored the complex relationships between these variables. LPA was conducted in Mplus 8.3 using the 10-item autistic traits scale to identify homogeneous subgroups. Models ranging from one to five profiles were estimated (see **Table 7**).

**Table 7:** Model indices for latent profile analysis of autistic traits.

Profiles	AIC	BIC	aBIC	LMR-LRT	BLRT	Entropy	1	2	3	4	5
1	57544.724	57658.067	57594.524	N/A	N/A	1	1				
2	46117.759	46293.440	46194.950	<0.001	<0.001	0.956	74.54	25.46			
3	41122.773	41360.794	41227.355	<0.001	<0.001	0.946	30.00	58.91	11.09		
4	39959.114	40259.474	40091.086	<0.001	<0.001	0.892	32.10	11.04	21.99	34.87	
5	38792.599	39155.298	38951.962	<0.001	<0.001	0.918	9.83	32.94	12.54	33.65	11.04

N/A, not applicable; AIC, Akaike information criterion; BIC, Bayesian information criterion; aBIC, adjusted Bayesian information criterion; LMR-LRT, Lo-Mendell-Rubin likelihood ratio test; BLRT, bootstrap likelihood ratio test.

As shown in the table, the information criteria (AIC, BIC, and aBIC) decreased progressively as the number of profiles increased. However, the 2-profile solution demonstrated the optimal fit. This model yielded a high Entropy value of 0.956, indicating excellent classification accuracy and clear differentiation between profiles. Both the LMR-LRT and BLRT were statistically significant (*p* < 0.001), and the smallest profile accounted for 25.46% of the sample, well above the 5% threshold for meaningful interpretation. Consequently, based on the fit indices and model parsimony, the 2-profile model was retained as the final solution.

**Figure 2:**
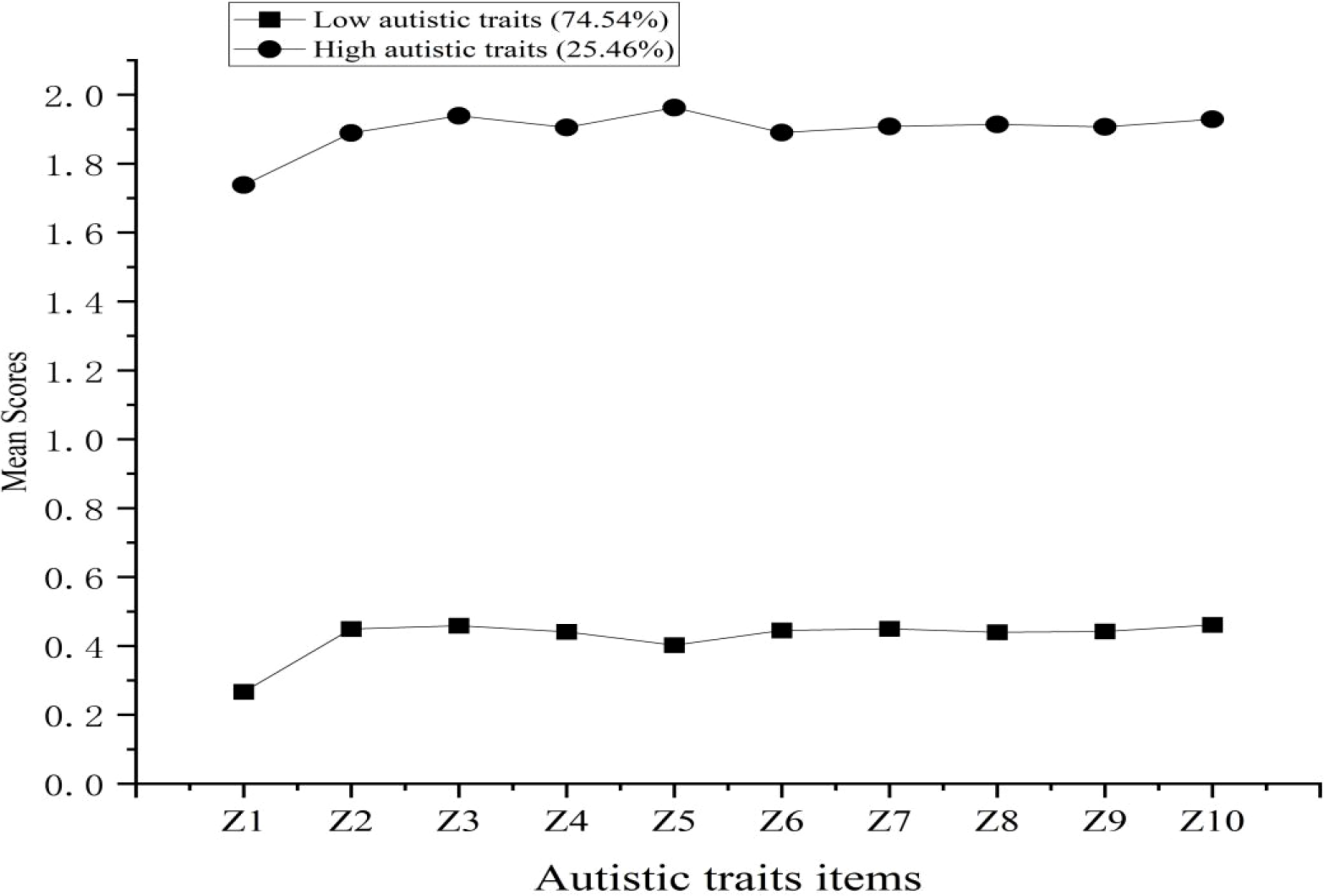
Mean scores and distribution of the two latent profiles of autistic traits. Z1–Z10 represent the ten items of the autistic traits scale.

**Figure 2** illustrates the characteristic response patterns of the two identified profiles based on the 10 autistic traits items. The first profile, comprising the majority of the sample (*N* = 1,593, 74.54%), was characterized by uniformly low scores across all items (ranging from approximately 0.2 to 0.5). Consequently, this profile was labeled as the “Low Autistic Traits” group. The second profile included 544 students (25.46% of the total sample). In contrast to the first group, this profile exhibited consistently higher scores on all indicators, with mean scores generally ranging from 1.7 to 2.0. Accordingly, this profile was labeled as the “High Autistic Traits” group. As shown in the figure, there is a clear distinction between the two profiles with no overlap in their response patterns.

**Table 8** presents the results of the Mann–Whitney U tests conducted to account for potential deviations from normality. The findings indicated that individuals in the High Autistic Traits profile exhibited significantly lower levels of physical activity (Z = -9.10, *p* < 0.001) and significantly higher levels of social anxiety (Z = -17.90, *p* < 0.001) compared to those in the Low Autistic Traits profile.

**Table 8:** Mann–Whitney U test results for differences in physical activity and social anxiety across autistic traits profiles (*N* = 2,137).

Predictor	*Profile*	*N*	Median	Mean rank	*Z*	*U*	Hodges-lehmann estimate	Rank-biserial correlation	95% CI
Physical activity	Low Autistic Traits	1593	36	1139.82	-9.10^***^	320500	12	-0.26	8,16
	High Autistic Traits	544	18	861.63					
Social anxiety	Low Autistic Traits	1593	9	929.65	-17.90^***^	655300	-6	0.51	-6,-5
	High Autistic Traits	544	14	1477.05					

***p<0.001.

**Table 9** further reports the results of independent-samples t-tests. Consistent with the nonparametric findings, individuals in the High Autistic Traits profile showed significantly lower physical activity (*t* = 7.91, *p* < 0.001, *d* = 0.39) and significantly higher levels of social anxiety (*t* = -23.25, *p* < 0.001, *d* = -1.16) compared to the Low Autistic Traits profile. Importantly, the results of the Mann–Whitney U tests and independent-samples t-tests were consistent in both direction and statistical significance, thereby confirming the robustness of the observed group differences.

**Table 9:** Independent-samples t-test results for differences in physical activity and social anxiety across autistic traits profiles (*N* = 2,137).

Predictor	Profile	*N*	*Mean*	*SD*	*t*	*Cohen’s d*	*95% CI*
Physical activity	Low Autistic Traits	1593	45.85	35.88	7.91^***^	0.39	10.46, 17.39
	High Autistic Traits	544	31.93	34.04			
Social anxiety	Low Autistic Traits	1593	9.19	5.50	-23.25^***^	-1.16	-9.59, -8.10
	High Autistic Traits	544	18.03	11.92			

***p<0.001.

## 4 Discussion

The present study integrated variable-centered and person-centered approaches to examine the relationships among physical activity, social anxiety, and autistic traits in female college students. Across these two complementary analytical perspectives, the findings consistently highlight the protective role of physical activity and the mediating function of social anxiety, thereby offering a more comprehensive understanding of the mechanisms underlying autistic traits in this population.

First, the results revealed a significant negative association between physical activity and autistic traits, supporting Hypothesis 1. This finding is consistent with prior research demonstrating that physical activity reduces social withdrawal, repetitive behaviors, and emotional dysregulation in both adolescent and clinical populations (73). Importantly, the present study extends these findings to a non-clinical sample of female college students, a group in which autistic traits are often more subtle and camouflaged.

From a theoretical perspective, this relationship can be understood through Bandura’s self-efficacy theory, which defines self-efficacy as individuals’ beliefs in their capability to organize and execute the behaviors necessary to achieve desired outcomes (74). Physical activity provides repeated opportunities for mastery experiences and social feedback, thereby enhancing individuals’ perceived competence and confidence in social contexts (75–77). As a result, individuals are better equipped to engage in interpersonal interactions and regulate their behavior, which may reduce the manifestation of autistic traits. In addition, prior research suggests that physical activity improves executive functioning and cognitive flexibility (78, 79), thereby mitigating rigid thinking patterns and stereotyped behaviors commonly associated with autistic traits.

Compared with previous studies that predominantly focused on clinical or male-dominated samples, the present findings suggest that the beneficial effects of physical activity are also evident in non-clinical female populations. This is particularly noteworthy given that female students often exhibit more socially adaptive yet internally strained forms of autistic traits. Thus, physical activity may serve as a compensatory mechanism that supports social adaptation in this group (80–82).

Second, the findings confirmed that social anxiety partially mediates the relationship between physical activity and autistic traits, supporting Hypothesis 2. This result is consistent with prior research indicating that physical activity reduces anxiety and promotes emotional regulation (83, 84). As social anxiety decreases, individuals are more likely to perceive social situations as less threatening and more controllable, which facilitates active engagement in social interactions.

Within the framework of social cognitive theory, emotional states play a central role in behavioral regulation (85, 86). Individuals experiencing high levels of social anxiety tend to adopt avoidance strategies, such as social withdrawal and reduced interpersonal engagement, which overlap with behavioral expressions of autistic traits. Conversely, lower social anxiety enhances individuals’ sense of control and flexibility in responding to social stimuli, thereby reducing maladaptive behavioral patterns. Furthermore, from the perspective of self-efficacy theory, reduced anxiety is associated with higher interpersonal self-efficacy, enabling individuals to process social cues more effectively and engage in adaptive social behaviors (87, 88).

Third, from a person-centered perspective, the study identified two latent profiles of autistic traits—”Low Autistic Traits” and “High Autistic Traits”—supporting Hypothesis 3. This finding differs from previous research reporting three- or four-class structures in mixed or clinical samples (59), suggesting that the structure of autistic traits may vary across samples. In the present study, the relatively homogeneous nature of female college students, along with the tendency for camouflaged traits, may have contributed to a simpler two-class structure.

Importantly, significant differences were observed between these profiles in terms of physical activity and social anxiety. Students in the “Low Autistic Traits” group reported higher levels of physical activity and lower levels of social anxiety. In contrast, those in the “High Autistic Traits” group exhibited the opposite pattern. These findings are consistent with the variable-centered results and further reinforce the interconnected roles of physical activity and social anxiety in shaping autistic traits (58).

In addition, the overall levels of physical activity and social adaptation among female college students were found to be moderate to low, which is consistent with previous findings (89). According to self-determination theory, the maintenance of health behaviors such as physical activity depends on the fulfillment of basic psychological needs, including autonomy, competence, and relatedness (90, 91). However, during the transition to emerging adulthood, female college students may experience disruptions in motivation due to academic pressure, identity exploration, and environmental constraints (92, 93). For instance, excessive academic demands and the widespread use of digital media may reduce opportunities for real-world social interaction and physical engagement (94, 95). Consequently, insufficient physical activity may limit opportunities to satisfy social relatedness needs, thereby hindering emotional regulation and increasing vulnerability to social anxiety and autistic traits.

Moreover, the distinct pattern observed in the “High Autistic Traits” group can be interpreted within the framework of social motivation theory (96). This theory posits that individuals possess an intrinsic drive to seek and derive reward from social interaction, and that deficits in this motivation system underlie autistic traits (97, 98). Individuals with elevated autistic traits exhibit reduced sensitivity to social rewards, which may diminish their motivation to engage in socially embedded activities such as group-based physical activity (99, 100).

Given that most university physical activity contexts inherently involve social interaction, these environments may be perceived differently by individuals with high autistic traits. Prior studies have shown that such individuals often experience social situations as cognitively demanding and emotionally taxing rather than rewarding (101, 102). Therefore, the social components of physical activity may function as sources of cognitive burden rather than motivation, which helps explain the lower participation levels observed in this group (103, 104).

Furthermore, the concept of withdrawal anxiety provides an additional explanatory framework. Withdrawal anxiety refers to a maladaptive cycle in which individuals, due to heightened anxiety in social contexts, engage in avoidance behaviors that reduce opportunities for positive social reinforcement and emotional regulation, thereby maintaining or exacerbating anxiety over time (105). In the present study, individuals in the “High Autistic Traits” group may be more likely to fall into this cycle, where social anxiety leads to reduced participation in physical activity, which in turn limits opportunities for emotional regulation and further intensifies anxiety.

Finally, these findings should also be interpreted within the Chinese cultural context. In many Chinese universities, physical activity is often organized in collective forms, such as group exercises or team sports, which emphasize coordination, cooperation, and social interaction (106, 107). While such environments may enhance social connectedness for most students, they may also increase perceived pressure for individuals with high autistic traits, particularly in a cultural context that values social harmony and conformity (19). Consequently, the effectiveness of physical activity as a protective factor may depend on the alignment between individual psychological characteristics and the social demands of the exercise context. Tailoring physical activity programs to include both group-based and individual formats may therefore enhance engagement and psychological benefits for diverse student populations.

## 5 Implications and limitations

Integrating variable-centered and person-centered perspectives, this study examines how social anxiety mediates the relationship between physical activity and autistic traits. Moreover, the study shows that female college students can be segmented into distinct subgroups according to differing presentations of autistic traits. These results offer fresh directions for research on autistic traits in non-clinical populations and carry important practical implications for preventing and intervening in autism-like behaviors among female college students.

First, higher education educators and mental health centers should prioritize cultivating physical activity habits among female college students and alleviating their social anxiety, fully recognizing the critical importance of physical activity and social adaptation for individual growth and development. Emerging adulthood, which encompasses the university phase, represents a critical period for developing lifelong physical activity habits, reshaping social cognition, and forming stable interpersonal interaction patterns. By helping female college students align their personal goals with campus demands, physical activity, combined with low social anxiety, fosters harmony between the self and the social environment, thereby promoting positive, autonomous development.

Next, to support the development of female college students, educators should prioritize helping them establish appropriate social and fitness goals and ensuring access to inclusive environmental resources that facilitate their growth. For example, by understanding the specific psychological needs of female college students, educators can help them clarify social values that align with both external environmental demands and their actual circumstances, thereby fostering realistic goal-setting, sustained engagement in exercise, and healthier social functioning. In particular, effectively reducing social anxiety hinges on helping students cultivate a strong “sense of social safety.”

Finally, educators should account for differences between groups. For female college students who exhibit different levels of autistic traits, universities can offer tiered psychological counseling and targeted group activities. For instance, universities could run small-group, sport-centered counseling sessions that provide low-pressure settings in which students with “High Autistic Traits” can progressively reduce social withdrawal and strengthen nonverbal communication. In addition, mental health curricula can be used to disseminate information about neurodiversity and social anxiety, enabling students to accurately understand and manage autistic traits and social anxiety.

This study is subject to several limitations. First, the present research employed a cross-sectional design. Since physical activity and social anxiety are variables that change over time, the cross-sectional nature of the data precludes rigorous determination of causal relationships among variables. Future research should adopt longitudinal designs to explore the dynamic development and causal pathways linking physical activity, social anxiety, and autistic traits.

Second, the investigation of mediating mechanisms remains limited. Although the mediating role of social anxiety was confirmed, other potential mediators may exist. Future studies should incorporate additional variables, such as sleep quality, body image, and peer support, to provide a more comprehensive understanding of the mechanisms underlying autistic traits among female college students.

Third, this study used convenience sampling, recruiting participants primarily from universities in a specific region. This non-random sampling strategy may limit the generalizability of the findings. Furthermore, as the sample consisted exclusively of female college students, the results cannot be generalized to male students or other age groups. Future research should include more diverse and representative samples, including different genders and clinical populations.

Fourth, there are limitations related to measurement. All variables were assessed using self-report questionnaires, which may introduce recall bias and common method variance. In particular, physical activity was measured using the PARS-3, which captures overall activity levels based on intensity, duration, and frequency, but does not distinguish between specific types of physical activity. Given that different forms of physical activity (e.g., individual vs. group-based) may have distinct psychological effects, future research should further differentiate among activity types. In addition, more objective measures, such as accelerometers, as well as clinical interviews for assessing autistic traits, are recommended to improve measurement accuracy and validity.

Finally, this study did not include certain potentially relevant demographic variables, such as academic major and family mental health history, and the sample was drawn primarily from urban universities. Future research should incorporate a broader range of control variables and recruit larger, multi-center samples to enhance the robustness and generalizability of the findings.

## 6 Conclusion

Based on a sample of 2,137 female undergraduates, this study integrated variable- and person-centered approaches to examine the mechanisms and heterogeneity of autistic traits. Results indicated that physical activity was negatively associated with autistic traits, with social anxiety partially mediating this relationship (31%). Latent Profile Analysis identified two subgroups—low and high autistic traits—with the high-trait group exhibiting lower physical activity and higher social anxiety.

These findings highlight physical activity and social anxiety as key, modifiable targets for intervention. Accordingly, higher education institutions should promote regular physical activity while addressing social anxiety, with particular emphasis on students with elevated autistic traits.

## Data Availability

The original contributions presented in the study are included in the article/supplementary material. Further inquiries can be directed to the corresponding authors.
